# Notum attenuates HBV-related liver fibrosis through inhibiting Wnt 5a mediated non-canonical pathways

**DOI:** 10.1186/s40659-019-0217-8

**Published:** 2019-03-13

**Authors:** Wenting Li, Xiaolan Yu, Chuanlong Zhu, Zheng Wang, Zonghao Zhao, Yi Li, Yonghong Zhang

**Affiliations:** 1 3rd Liver Unit, Department of Infectious Disease, Anhui Provincial Hospital, Hefei, People’s Republic of China; 2 Department of Ear-Nose-Throat, Anhui Provincial Hospital, Anhui Medical University, Hefei, People’s Republic of China; 3 Department of Infectious Disease, The First Affiliated Hospital of Nanjing Medical University, Nanjing, People’s Republic of China; 4 Department of Respiratory and Critical Medicine, People’s Hospital of Zhengzhou University, Zhengzhou, People’s Republic of China; 5 Department of General Surgery, The First Affiliated Hospital of Anhui Medical University, Hefei, People’s Republic of China; 6 Anhui Provincial Hospital, Anhui Medical University, Hefei, 230001 Anhui People’s Republic of China; 7 The First Affiliated Hospital of USTC, Division of Life Sciences and Medicine, University of Science and Technology of China, Hefei, 230001 Anhui People’s Republic of China

**Keywords:** Notum, Wnt signaling pathway, HBV, Liver fibrosis

## Abstract

**Background:**

Non-canonical Wnt pathways play important roles in liver fibrosis. Notum is a newly discovered inhibitor to Wnt proteins. This study was to investigate anti-fibrotic effects of Notum.

**Methods:**

53 patients with hepatitis B virus (HBV) infection as well as a cell co-culture system of LX-2 and Hep AD38 cells were engaged in this study. Clinical, biological and virological data of each patient were analyzed. Cell viability was detected at different time points. mRNA and protein levels of NFATc1 (Nuclear factor of activated T-cells), Jnk, α-SMA, Col1A1 and TIMP-1 were detected both in LX-2 and liver tissue. Protein levels of NFATc1 and Jnk in liver tissue and their correlations with fibrosis score were analyzed.

**Results:**

Hepatitis B virus replication up-regulated Wnt5a induced NFATc1 and Jnk activity in Hep AD38. Notum suppressed NFATc1, Jnk and fibrosis genes expression, reduced cell viability in co-cultured LX-2 cells induced by HBV. Interestingly, Patients with HBV DNA > 5log copies/ml had higher mRNA levels of NFATc1 and fibrosis genes than patients with HBV DNA < 5log copies/ml. Most importantly, protein expressions of NFATc1 and pJnk have positive correlations with liver fibrosis scores in HBV-infected patients.

**Conclusions:**

Our data showed that Notum inhibited HBV-induced liver fibrosis through down-regulating Wnt 5a mediated non-canonical pathways. This study shed light on anti-fibrotic treatment.

## Background

Liver fibrosis was considered to be a wound-healing response of liver to chronic liver injuries including alcohol, hepatitis and immune compound. It was characterized by an excessive deposition of extracellular matrix (ECM) components in liver [1]. So far, activated hepatic stellate cell (HSCs), the main source of accumulated ECM, has been evidenced to be the predominant cell type responsible for liver fibrosis development. Thus, repressing HSC activation may prevent and reverse liver fibrosis [2].

Hepatitis B virus (HBV) infection is a worldwide health problem with nearly 400 million carries worldwide [3]. Chronic infection resulted in liver fibrosis and following cirrhosis or even liver cancer. If treated properly at an early stage, HBV-induced liver fibrosis can be reversed [4, 5]. Unfortunately, the mechanism of HBV-related liver fibrosis remains poorly understood.

Among various signaling pathways and cytokines involved in liver fibrosis, Wnt signaling is considered to be the most important one [6]. Wnt pathways can be mainly divided into canonical and non-canonical pathway according to the cascades it evokes [7]. For a long time, Wnt/β-catenin (canonical) pathway has been considered to be of vital importance in HSC activation and liver fibrosis [8]. However, with the further understanding of the role of Wnt pathway in liver fibrosis, abnormal activation of non-canonical pathways mediated by Wnt 5a through Jnk and NFAT have been tightly associated with HSC activation and liver fibrosis development [9–11].

Notum, a secreted protein, was reported to deacylate various Wnt proteins leading to the inhibition of both canonical and non-canonical pathways simultaneously [12, 13]. Very few data showing the association between Notum and liver fibrosis are available. This study was designed to explore the anti-fibrosis effect of Notum in LX-2 cells and patients with chronic HBV infection.

## Materials and methods

### Materials

0.4-μM Transwells were bought from Costar Corp. (USA). Serum levels of alanine transaminase (ALT), aspartate transaminase (AST), lactic dehydrogenase (LDH) in patients were determined by routine laboratory tests in Anhui Provincial Hospital (Hefei, China). Kits for serum Hydroxyproline (Hyp) was bought from Nanjing Jiancheng Bioengineering Institute (Nanjing, China). Recombinant Human Wnt 5a was bought from R&D Systems (645-WN-010/CF). Viability of Hepatic stellate cell line (LX-2) cells was detected by Cyto Tox 96 Nonradioactive Cytotoxicity assay from Promega (USA). The primary antibodies were as following: rabbit anti-Jnk (#9252, Cell signaling), rabbit anti-pJnk (Thr 183/Tyr 185, #4668, Cell signaling), mouse anti-α-SMA (ab5694, Abcam), mouse anti-NFATc1 (sc-7294, Santa Cruz), rabbit anti-Collagen 1A1 (MA1-141, ThermoFisher Scientific, USA), mouse anti-β-actin (ab8227, Abcam). Horseradish peroxidase conjugated secondary antibodies were obtained from GE Healthcare (UK).

### Cell culture and viral stocks

Hepatic stellate cell line (LX-2) and Hep AD38 cell line were purchased from Cancer Institute and Hospital, Chinese Academy of Medical Sciences. Cells were maintained in Dulbecco’s modified Eagle’s medium (DMEM, Invitrogen, California, USA) containing 10% new bovine serum (Hangzhou, China), 100 U/ml penicillin and 100 μg/ml streptomycin, under a humidified 5% (v/v) CO_2_ atmosphere at 37 °C. Hepatitis B virus used in this study was collected from the supernatant of Hep AD38 cells.

Entecavir (ETV) was diluted into serum free DMEM at a final concentration of 500 nM. At 24, 48 and 72 h after treatment, supernatant of Hep AD38 was collected and cells were harvested for RNA and DNA extraction and HBV DNA level detection.

### Plasmid pCMV6-AC-GFP-Notum preparation and transfection

Expression plasmid pCMV6-AC-GFP-Notum driven by a CMV promoter was bought from Origene (NM_178493, USA). The plasmid pCMV6-AC-GFP-Notum was purified, resuspended in double-distillation H_2_O (ddH2O) with a concentration of 1 μg/μl and stored at − 20 °C.

LX-2 cells were cultured in 6-well plates until 50–80% confluence. Diluted into serum-free DMEM, pCMV6-AC-GFP-Notum or pCMV6-AC-GFP-neo plasmids were mixed with Lipofectamine LTX reagent (Life Technologies) according to the manufacturer’s instructions. After incubation at room temperature for 5 min, the solution was added to one of duplicated wells with HSCs, and transfection was performed at a humidified 5% (v/v) CO_2_ atmosphere at 37 °C for 12 h.

### Co-culture of Hep AD38 and LX-2 cells in a transwell system

A co-culture transwell system of Hep AD38 and LX-2 cells was used to imitating cell interaction during HBV-induced liver fibrosis. The system was performed as follows: Hep AD 38 cells were seeded at 2 × 10^5^ cells/well into 12-well plates, and LX-2 cells were seeded at 1 × 10^5^ cells/well on the membrane of Transwells. Cultured overnight, transwells were stacked into Hep AD38 containing wells. At 72 h after incubation, cells were harvested for RNA and Protein collection.

### Cell viability determination

At 72 h after transfection, LX-2 cells were collected for cell viability assay. Cells were seed in 96-well plate at the density of 1 × 10^4^ cells/ml in DMEM supplemented with 10% fetal bovine serum for 12, 24, 48 h. Transfection of pCMV6-AC-GFP-Notum was observed by immunofluorescence microscope. Cell viability was measured by CytoTox 96 Nonradioactive Cytotoxicity assay (Promega) by testing the lactate dehydrogenase activity according to the manufacturer’s instructions. The viability of other cells was measured via the same process.

### ELISA

For evaluation of secreted Wnt 5a, Hep AD 38 cells were seeded into 24 well plate at a density of 5 × 10^4^ cell/ml. After that, cells were treated with 500 nM Entecavir for 24, 48 and 72 h. In the end, cell culture supernatants were collected and stored at − 80 °C. The concentration of Wnt 5a was detected by ELISA according to the manufacturer’s instructions. The absorbance was read by AlphaScreen Compatible Microplate Reader (Bio Tek Synergy2, USA). The limit of sensitivity of Wnt was < 10 ng/ml. Samples were run in triplicate.

### Patients

From April 2014 to August 2016, 95 adult patients with chronic hepatitis B were identified at Anhui Provincial Hospital (Hefei, China) according to the criteria established by the Chinese Medical Association. These patients were nucleotide analogue and interferon treatment naive. On the contrary, 42 patients were excluded from this study owing to severe heart, lung or renal diseases and other chronic liver disease, especially HBV/HCV/HIV co-infection as well as previous nucleotide analogue or interferon treatment. In addition, seven patients without HBV/HCV/HIV infection were enrolled as control. All patients in this study underwent liver biopsy. Data from patients in this study were analyzed according to liver fibrosis stage or serum HBV load, that is, series 1: Control, G_0–1_S_0–1_, G_2_S_2_, G_3_S_3_, G_4_S_4_; series 2: Control, HBV DNA level < 5log copies/ml, HBV DNA level > 5log copies/ml. These patients gave written informed consent for this study, which was approved by the ethics committee of Anhui Provincial Hospital.

### Biochemical examinations and hepatitis B virus DNA quantification

Liver function of patients in this study including serum alanine transaminase (ALT), aspartate aminotransferase (AST), total bilirubin (TBil) and albumin (ALB) levels were measured by routine laboratory tests by 7170-automatic biochemistry analyzer (Tokyo, Japan). Moreover, serum hepatitis B virus DNA levels were measured by quantitative PCR assay (COBAS TaqMan HBV tset).

### Histopathological examination

Liver tissue of these patients in this study were collected by liver biopsy using Bard biopsy needle (18 G, 910 cm; USA) as previously described [14]. Liver tissue with a length of 15 mm was fixed in 10% formalin, embedded in paraffin and sectioned at a thickness of 5 μm. Haematoxylin–eosin (H&E) staining and Masson’s trichrome staining were used to examine the changes in liver pathology and collagen deposition, respectively. The hepatic fibrosis score was measured by two independent pathologists blindly according to METAVIR system [15]: S0, no scarring; S1, minimal scarring; S2, extended scarring; S3, bridging fibrosis; S4, cirrhosis or advanced scarring of the liver.

### Immunohistochemistry

Immunohistochemical staining was performed on 5 μm thickness paraffin-embedded liver tissue sections to determine the expression and distribution of NFATc1 and Jnk.

Briefly, after blockage with endogenous peroxidase and bovine serum albumin, the liver sections were incubated with NFATc1 or Jnk antibody at 4 °C over night, respectively. The sections were then incubated with HRP-labelled goat-anti-rabbit or goat-anti-mouse secondary antibodies (diluted to 1:200) at 37 °C for 1 h. The expressions of NFATc1 and Jnk were analyzed by light microscope (Olympus, Tokyo, Japan). Data were presented as a PI. Positive index (PI) = mean optical density × positive area percentage.

### Reverse transcriptase-polymerase chain reaction (RT-PCR) and real-time PCR

Liver tissue sections with 2–3 mm long was immediately stored in RNA Later (Invitrogen) for RNA detection after liver biopsy. Total RNA were extracted from liver tissue and cells by RNeasy Lipid Tissue Mini Kit and RNeasy Mini Kit according to the manufacturer’s protocol (Qiagen, Germany), respectively. cDNA was synthesized from total RNA by RT-PCR using ReverTra Ace qPCR RT Kit (Toyobo, Shanghai, China) following the manufacturer’s protocol.

Real-time PCR was performed using SYBR Green Real-time PCR Master Mix (Toyobo) by ABI 7500 PRISM (Applied Biosystems Inc., USA). Target cDNA expression was quantified by comparing ∆∆Ct to GAPDH which was used as an internal control. The primers were listed in Table 1.

**Table 1:** List of primers used in qRT-PCR analysis

Target	Species	Forward 5′–3′	Reverse 5′–3′
α-SMA	Human	AAAAGACAGCTACGTGGGTGA	GCCATGTTCTATCGGGTACTTC
TIMP-1	Human	ACTTCCACAGGTCCCACAAC	GCTAAGCTCAGGCTGTTCCA
Col 1A1	Human	CAGCCGCTTCACCTACAGC	TCAATCACTGTCTTGCCCCA
NFATc1	Human	CAACGCCCTGACCACCGATAG	GGCTGCCTTCCGTCTCATAGT
Wnt 5a	Human	GGACCACATGCAGTACATCG	CCTGCCAAAAACAGAGGTGT
GAPDH	Human	ACCTTCCCCATGGTGTCTGA	GCTCCTCCTGTTCGACAGTCA

### Western blot assays

Protein was extracted from cells according to the instructions provided in the kits (Qiagen, Germany). At 72 h after transfection, cells were harvested and prepared for protein extraction immediately. The extracted proteins were separated by running on a 10% SDS-PAGE gel and transferred electrophoretically onto polyvinylidene fluoride membranes (PVDF). After incubation with 5% BSA, the membranes were incubated with anti- NFATc1 or pJnk antibody (1:1000) overnight at 4 °C. After being washed with TBST solution [Tris 10 mM, NaCl 150 mM, Tween-20 0.05% (V/V)], the blots were incubated with HRP-labeled goat-anti-rabbit secondary antibodies for 1 h. After that, the blots were washed by TBST again and then colored by ECL. The relative expressions were quantified by comparing band with β-actin.

### Statistical analyses

All data were represented as mean ± SEM. Statistical analyses were conducted using one-way analysis of variance (ANOVA) and SNK-q test with the SPSS 17.0 statistical software package. *P* < 0.05 was considered to be statistically significant.

## Results

### Notum suppressed Wnt 5a induced LX-2 cell activation

**Fig. 1:**
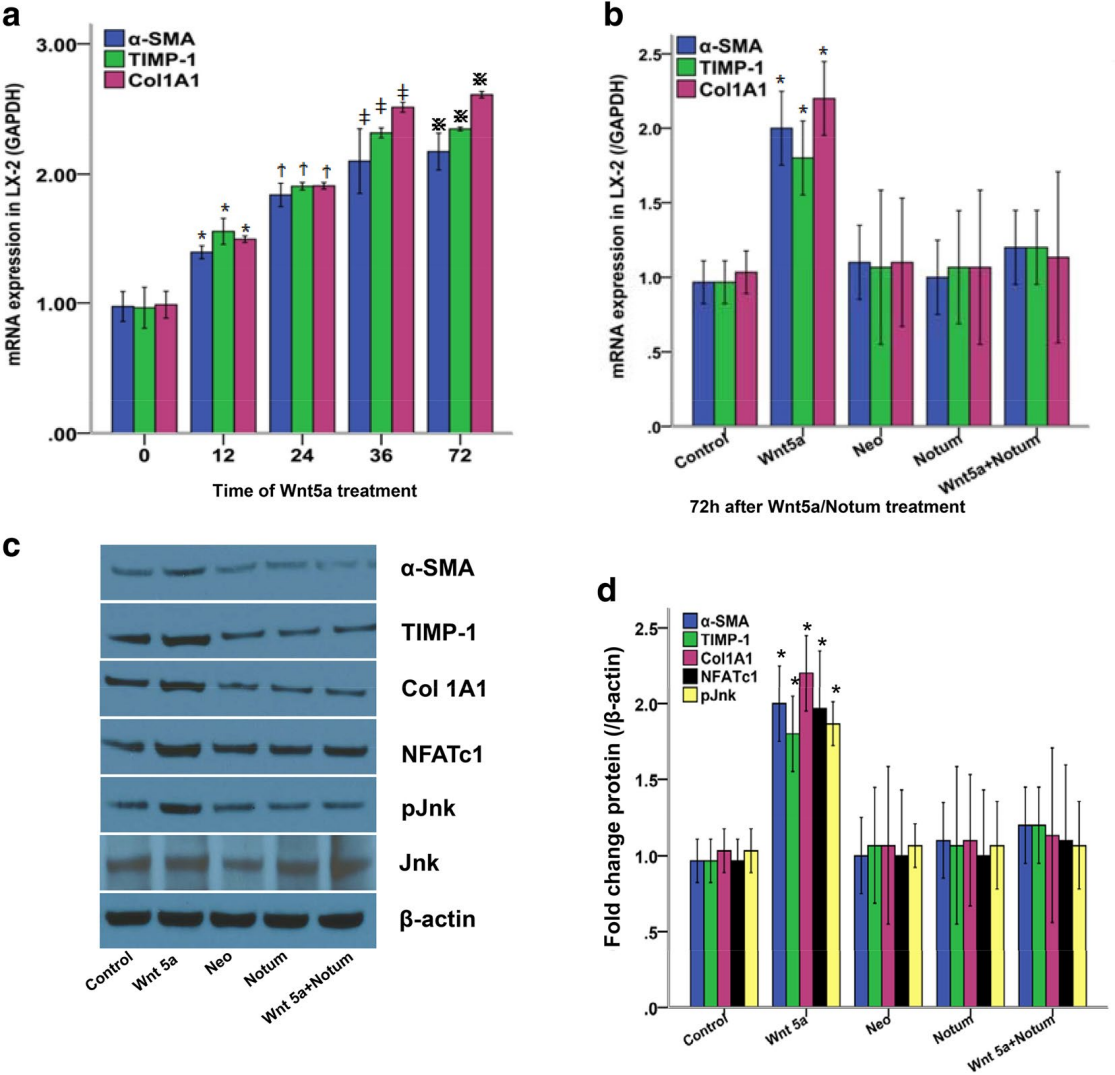
Notum inhibited Wnt 5a induced LX-2 cell activation. **a** 100 ng/ml Wnt 5a treatment enhanced LX-2 activation at a time-dependent manner, peaking at 36 h; *P < 0.05, ^Ϯ^*P *< 0.05, ^ǂ^*P *< 0.05, ^※^*P *< 0.05, compared with 0 h;, ^※^*P *> 0.05, compared with **ǂ**. **b**–**d** pCMV6-AC-GFP-Notum was transfected into LX-2 cells. All cells were incubated with 100 ng/ml Wnt 5a for 72 h. **b** α-SMA, TIMP-1 and Col 1A1 mRNA expressions in LX-2 cells treated with Notum/Wnt 5a, *P < 0.05. **c**, **d** Protein expressions of α-SMA, TIMP-1, Col 1A1, NFATc1 and pJnk in LX-2 cells treated with Notum/Wnt 5a. The data are derived from three independent experiments

Wnt 5a has been reported to mediate both canonical and non-canonical Wnt pathways. We first determined whether Wnt 5a protein could induce LX-2 cell activation. As shown in Fig. 1a, 100 ng/ml Wnt 5a treatment significantly enhanced the mRNA and protein expressions of α-SMA and Col 1A1 on LX-2 cells compared with normal cells, peaking at 36 h (*P *< 0.05, respectively).

Furthermore, we transfected pCMV6-AC-GFP-Notum into LX-2 cells to observe whether Notum could inhibit Wnt 5a induced LX-2 activation. As shown in Fig. 1b–d, 72 h incubation with 100 ng/ml Wnt 5a significantly up-regulated mRNA and protein expressions of α-SMA, TIMP-1 and Col1A1 in LX-2 cells compared with control group (*P *< 0.05, respectively), while they were significantly reduced by Notum compared with Wnt 5a treatment group (*P *< 0.05, respectively). Furthermore, we detected the protein expression of Jnk and pJnk in LX-2 cells. As a result, Wnt 5a treatment had no effect on Jnk protein expression while increased Jnk phosphorylation in LX-2 cells compared with control group (Fig. 1c, d, *P *< 0.05, respectively). Not surprisingly, Notum decreased Jnk phosphorylation in LX-2 cells induced by Wnt5a (*P *< 0.05, respectively).

### HBV replication contributed to Wnt 5a expression in Hep AD38 cells

**Fig. 2:**
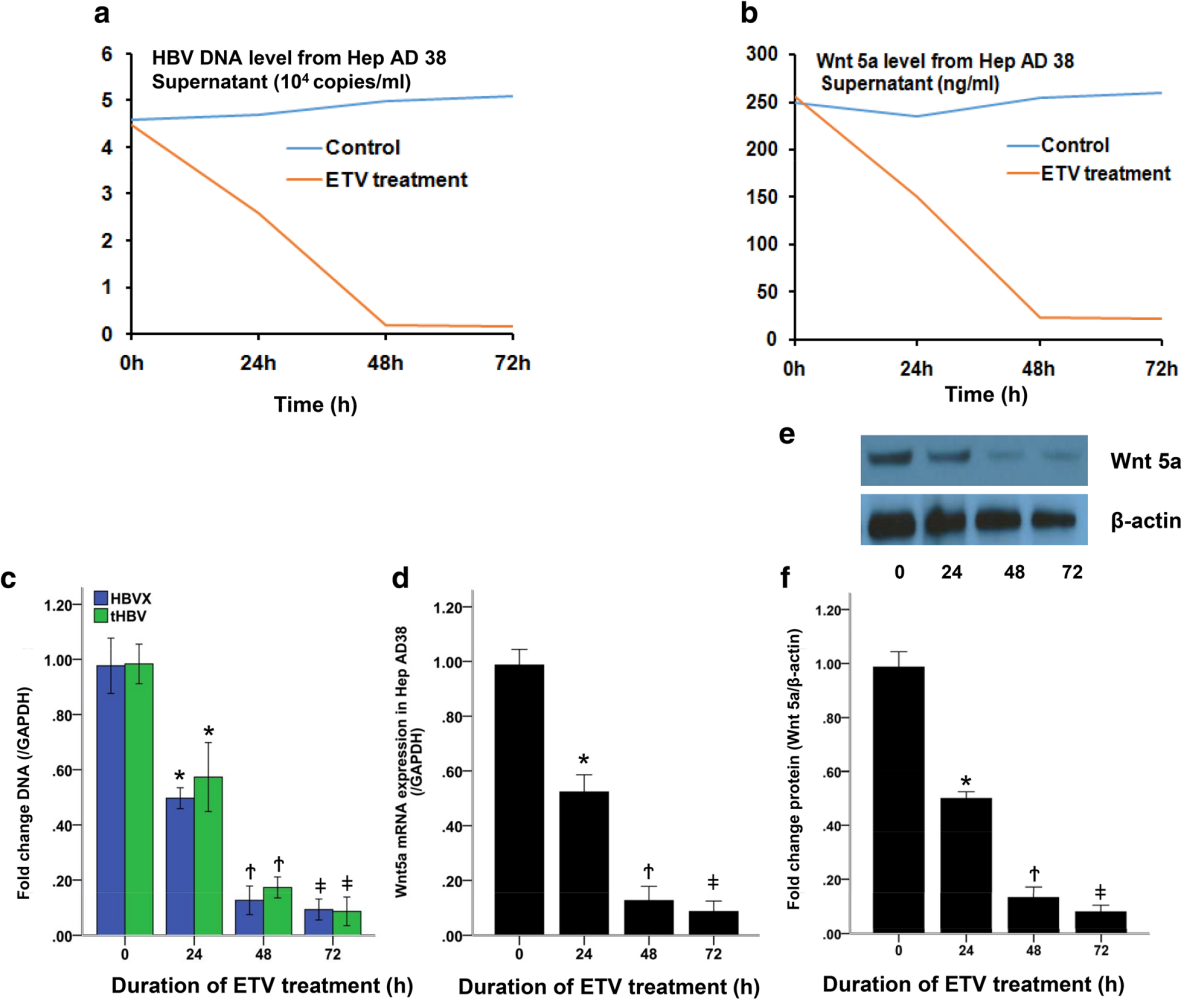
ETV reduced HBV replication and Wnt5a expression in Hep AD38 cells. Cells were treated with ETV at a concentration of 500 nM for 72 h. **a** HBV DNA level in supernatant was detected by qPCR; **b** Wnt 5a level in supernatant was detected by ELISA; **c** HBV X and HBV DNA levels in Hep AD38 cells were detected by qPCR; **d** Wnt 5a mRNA level in Hep AD38 cells was detected by qPCR; **e**, **f** Wnt 5a protein level in Hep AD38 cells was detected by Western-blot. *P < 0.05, ^Ϯ^*P *< 0.05, ^ǂ^*P *< 0.05, compared with 0 h; ^ǂ^*P *> 0.05, compared with Ϯ. All data were from three individual experiments

In order to explore the relationship between HBV replication and Wnt 5a, we inhibited HBV DNA replication by Entecavir (ETV) and detected Wnt 5a level from the supernatant of Hep AD38 cells by qPCR and ELISA, respectively. As shown in Fig. 2a, ETV reduced HBV DNA level in supernatant of Hep AD38 cells, which reached its valley at 48 h after ETV treatment. Interestingly, Wnt 5a level also went down under ETV treatment, which reached its lowest point at 48 h, similarly (Fig. 2b).

To further investigate the effects of HBV replication on Wnt 5a pathway, we tested mRNA and protein expressions of Wnt 5a as well as HBV replication in Hep AD 38 cells. As shown in Fig. 2c, ETV suppressed HBV X expression and HBV DNA replication in Hep AD38 cell. As a result, both mRNA and protein expressions of Wnt 5a were reduced by ETV treatment (Fig. 2d, e; *P *< 0.05, respectively).

### Notum inhibited HBV-induced liver fibrosis gene expression

**Fig. 3:**
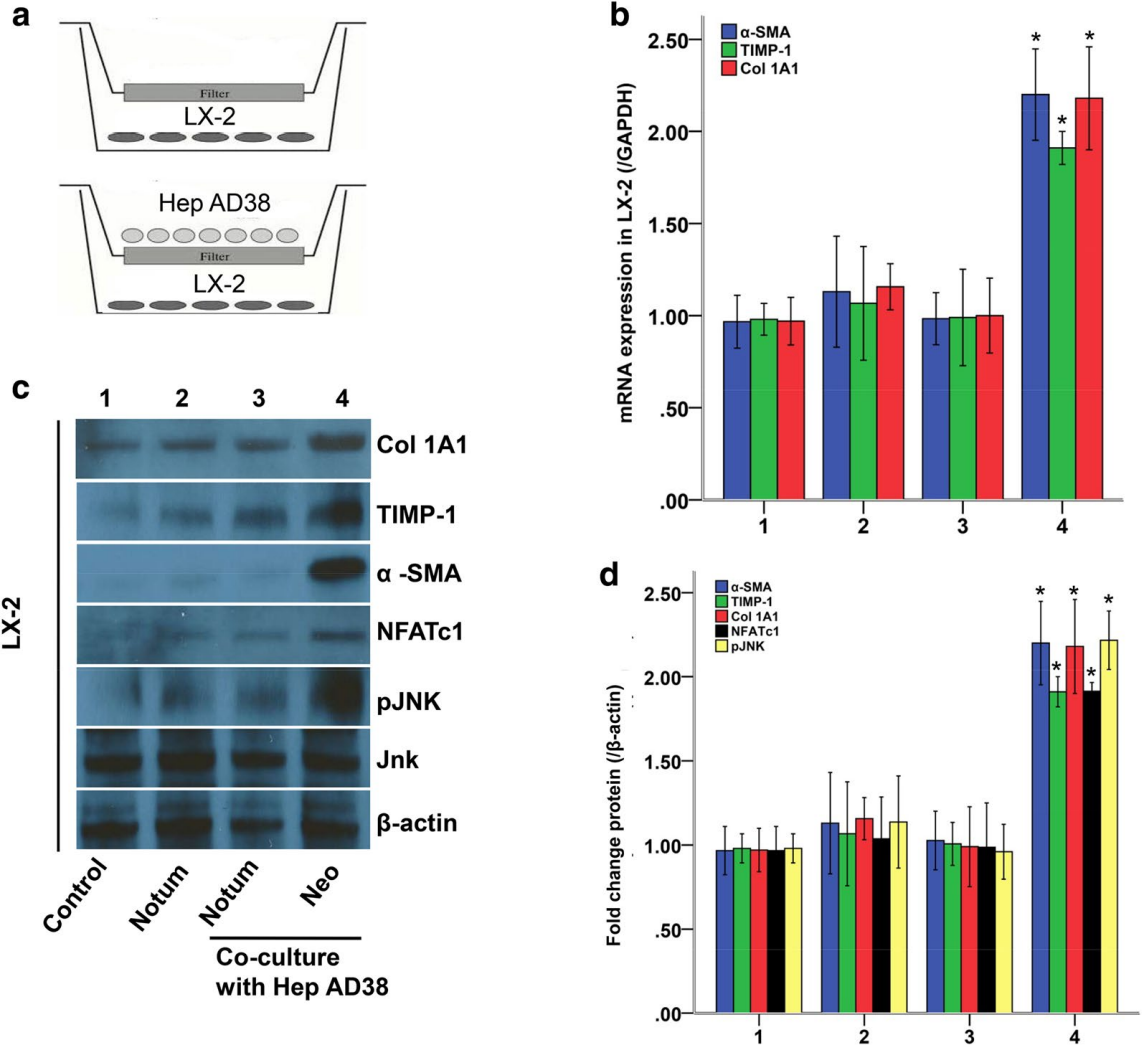
Notum inhibited liver fibrosis gene expression in LX-2 cells with HBV exposure. **a** Cell co-culture model. **b** LX-2 cells were co-cultured with Hep AD38 cells and transferred with Notum. α-SMA, TIMP-1 and Col 1A1 mRNA expressions in LX-2 cells were measured by qPCR. **c**, **d** α-SMA, TIMP-1 and Col 1A1 protein expressions in LX-2 cells were measured by Western-blot. *P < 0.05 compared with mock. All experiments were tried 3 times

In order to elucidate the mechanism of HBV-induced liver fibrosis, we observed the interaction between LX-2 and Hep AD 38 cells using a cell co-culture model (Fig. 3a). Using this model, we measured the expressions of fibrosis related genes and Wnt signaling activation in LX-2 cells when exposed to HBV. As a result, We again found that there were higher mRNA (Fig. 3b) and protein (Fig. 3c) expressions of NFATc1, Jnk, α-SMA, TIMP-1 and Col1A1 in LX-2 cells co-cultured with Hep AD38 than control LX-2 cells, while they were all sharply reduced by Notum treatment (*P *< 0.05, respectively).

### General data of patients with chronic hepatitis B

Furthermore, 53 HBV-infected patients were enrolled in this study to explore the role of Wnt pathway in HBV-induced liver fibrosis, seven patients with no hepatitis infection as control, shown in Table 2. The clinical, biological, virological and pathological data of each patient were analyzed. As a result, serum levels of AST, ALT, total bilirubin and HBV DNA were all significantly higher than control (*P *< 0.05, respectively). More importantly, the grade of liver inflammation and scores of liver fibrosis were both higher than control (*P *< 0.05, respectively).

**Table 2:** The clinical, biochemical and laboratory data of 60 patients

Characterize	Control	Patients	*P*
Gender (M/F)	3/4	25/28	> 0.05
Age (years)	29.2 ± 9.9	30.1 ± 10.2	> 0.05
Liver inflammation (G :1/2/3/4)	5/2/0/0	4/16/23/10	
Liver fibrosis (S: 1/2/3/4)	4/3/0/0	6/18/20/9	
ALT (IU/l)	21.2 ± 10.2	64 ± 21.1	< 0.05
AST (IU/l)	18.8 ± 8.3	51.3 ± 9.8	< 0.05
Total bilirubin (μmol/l)	7.2 ± 2.3	27.8 ± 11.1	< 0.05
Albumin (g/l)	48.1 ± 15.3	36.3 ± 12.6	< 0.05
HBV DNA (log copy/ml)	0	4.7 ± 1.5	< 0.05

### Liver pathology and immunohistochemical staining

Liver fibrosis/inflammation status was evaluated by H&E and Masson’s staining. Generally, liver tissue samples from control group (G_0–1_S_0–1_) showed normal lobular architecture with regular hepatic cord pointing to the central veins (Data were not shown). On the contrary, liver tissue samples from fibrosis group (G_2_S_2_, G_3_S_3,_ G_4_S_4_) showed more inflammatory cells infiltration and collagen deposition (Data were not shown). The liver structure was destroyed and there were much more necrotic and apoptotic cells in G_4_S_4_ group than in control group (Data were not shown). In addition, in the other fibrosis group (G_0–1_S_0–1_, G_2_S_2_, G_3_S_3_), inflammatory cell infiltration, hepatocyte necrosis and apoptosis were all remarkably reduced and collagen deposition was obviously reduced compared with G_4_S_4_ group (Data were not shown).

**Fig. 4:**
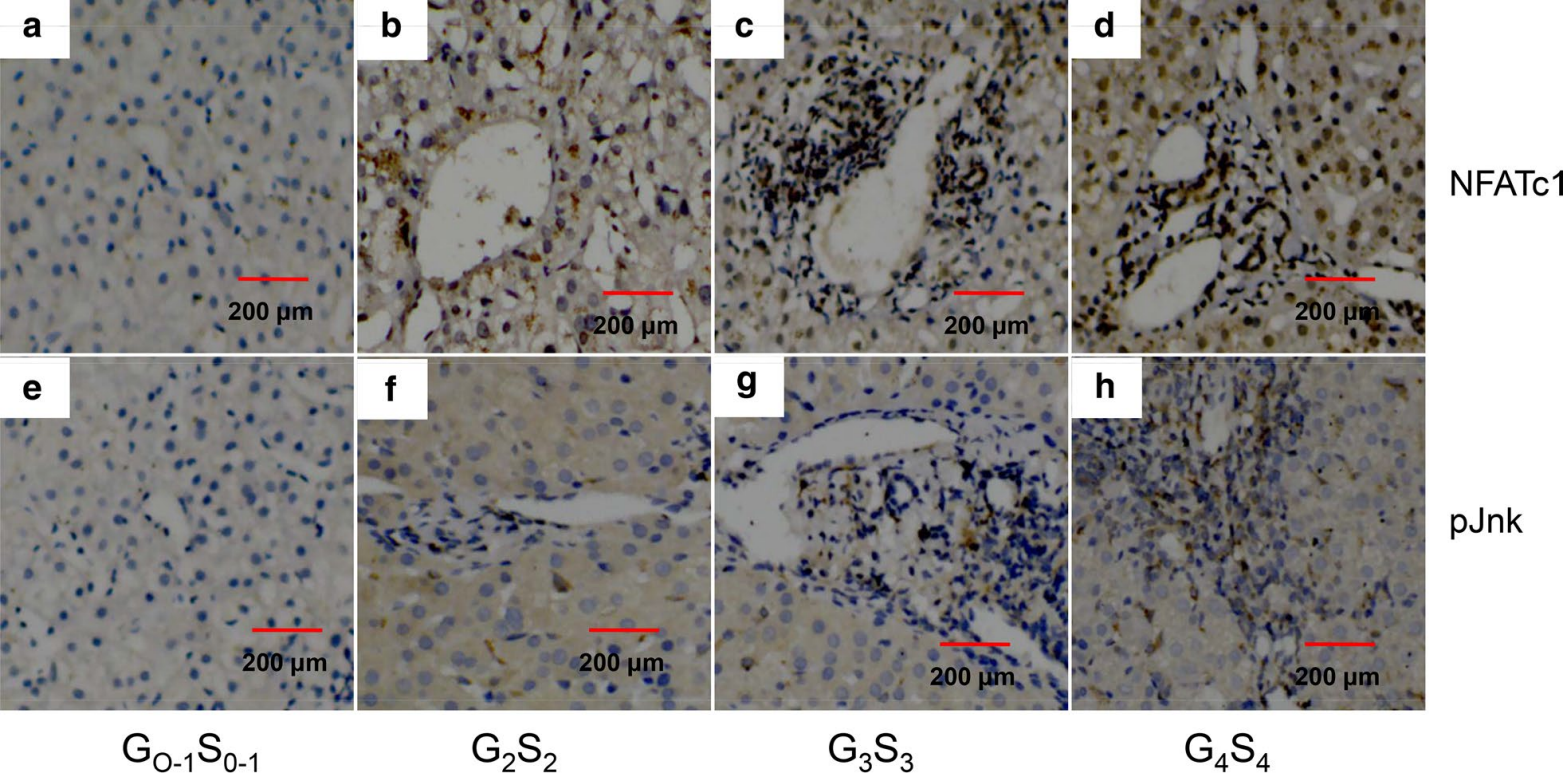
Expression of NFATc1 and pJnk in liver tissue. NFATc1 (**a**, **b**, **c**, **d**) and pJnk (**e**, **f**, **g**, **h**) expression were measured by immunohistochemical staining (×200). The intensity of expression was evaluated by Positive index (PI) = mean optical density × positive area percentage. NFATc1 and pJnk expression in liver tissue were analyzed according to liver fibrosis score. **a** vs **b** vs **c** vs **d**: 0.040 ± 0.009 vs 0.266 ± 0.087 vs 0.700 ± 0.094 vs 0.835 ± 0.052; *P* < 0.05, respectively. **e** vs **f** vs **g** vs **h**: 0.045 ± 0.014 vs 0.195 ± 0.067 vs 0.596 ± 0.044 vs 0.749 ± 0.088; *P* < 0.05, respectively

The expression and distribution of NFATc1, Jnk in liver tissue of patients was detected by immunohistochemical staining. As shown in Fig. 4, there were much more NFATc1, pJnk positive regions around the peri-portal fibrotic band areas, central vein and fibrous septa in liver tissue of patients with G_4_S_4_ compared with G_0–1_S_0–1_ (Fig. 4d vs a, h vs e).

### mRNA and protein expression of NFATc1 and Jnk in liver tissue

**Fig. 5:**
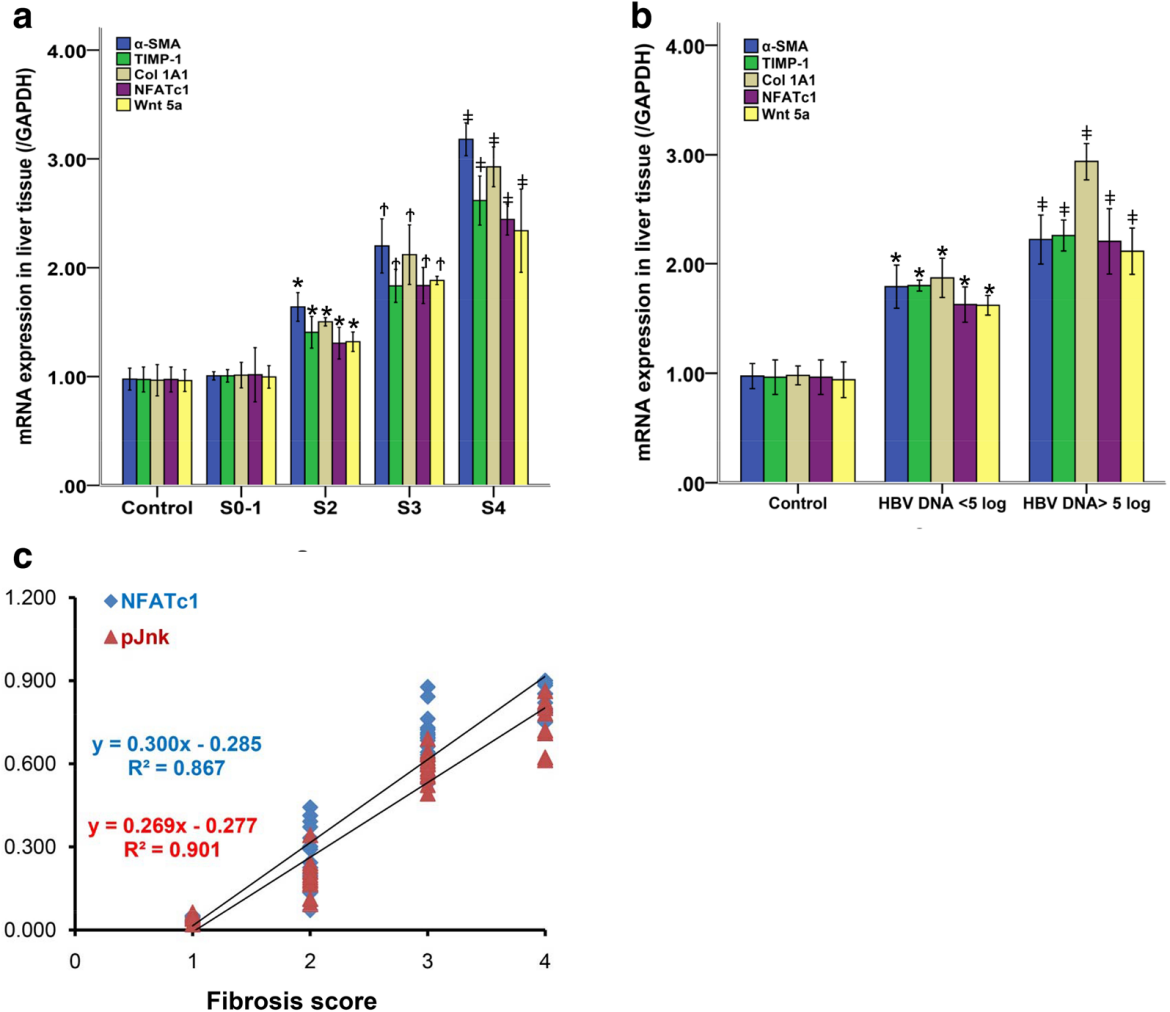
mRNA expressions of α-SMA, TIMP-1, Col 1A1, NFATc1 and Wnt 5a in liver tissue. **a** mRNA expressions of NFATc1, Wnt 5a and fibrosis genes in tissue was measured by qPCR in HBV-infected patients with different liver fibrosis score; *P < 0.05, ^Ϯ^*P *< 0.05, ^‡^*P *< 0.05, compared with control. **b** mRNA expressions of NFATc1, Wnt 5a and fibrosis genes in tissue by qPCR in HBV-infected patients with different viral load; *P < 0.05, ^‡^*P *< 0.05, compared with control. **c** NFATc1, pJnk protein expressions in liver tissue was measured by immunohistochemistry and positive index (PI). The relationship between NFATc1, pJnk protein expressions and liver fibrosis was observed by correlation analysis. For NFATc1, r = 0.931, *P *< 0.05; For pJnk, r = 0.950, *P* < 0.05. All data presented here were from three independent experiments

The mRNA expression of NFATc1 in patients was detected by real-time PCR and the expression of NFATc1 and Jnk protein was evaluated by light microscope. As a result, NFATc1 mRNA expression sharply increased in liver tissue from 53 HBV-infected patients compared with seven controls (*P* < 0.05). As shown in Fig. 5a, the mRNA level of Wnt 5a, NFATc1, α-SMA, Col 1A1 and TIMP-1 in liver tissue of patients with an S3/4 fibrosis score was much higher than in those with S1/2 (*P *< 0.05, respectively). Furthermore, indicated by Fig. 5b, according to the serum HBV DNA level, the mRNA level of Wnt 5a, NFATc1, α-SMA, Col 1A1 and TIMP-1 in liver tissue of patients with HBV DNA > 5log copies/ml increased compared to those with HBV DNA < 5log copies/ml (*P *< 0.05, respectively).

Moreover, the protein expressions of NFATc1 and pJnk in liver tissue were assessed by the positive indexes (PI). As a result, PI of NFATc1 and pJnk went up sharply along with the progression of liver fibrosis/inflammation status (*P *< 0.05, respectively). PI of NFATc1 were as follows: G_0–1_S_0–1_, 0.040 ± 0.009; G_2_S_2_, 0.266 ± 0.087; G_3_S_3_, 0.700 ± 0.094; G_4_S_4_, 0.835 ± 0.052; respectively. PI of pJnk were as follows: G_0–1_S_0–1_, 0.045 ± 0.014; G_2_S_2_, 0.195 ± 0.067; G_3_S_3_, 0.596 ± 0.044; G_4_S_4_, 0.749 ± 0.088; respectively. Therefore, NFATc1 and Jnk protein expression also rose as liver fibrosis and inflammation progress.

### NFATc1 and Jnk expression had positive correlations with the degree of liver fibrosis in CHB patients

The association between NFATc1 and Jnk expression and the degree of liver fibrosis was also assessed by correlation analysis. Not surprisingly, as showed by Fig. 5c, both NFATc1 and pJnk expression positively correlated with the degree of liver fibrosis: NFATc1 protein expression, r = 0.931, *P *< 0.05; pJnk protein expression, r = 0.950, *P* < 0.05.

## Discussion

Liver fibrosis has long been considered as a wound-healing response of liver to chronic liver injuries, including hepatitis, immune compounds, alcohol and so on. HBV remains the major cause of liver fibrosis and cirrhosis in China, an epidemic area. Although anti-viral therapy attained certain success, chronic HBV infection may also inevitably progress to liver fibrosis and even cirrhosis. However, HBV induced liver fibrosis is a complicated process, which is associated with hepatocyte, macrophage, stellate cell and various signaling pathways as well.

Among various pathways involved in liver fibrosis, Wnt signaling pathway has been regarded as a most important one. Wnt induced transcription activity mainly through two pathways, the canonical pathway and non-canonical pathway. Cumulative evidence has demonstrated the correlation of non-canonical Wnt pathway and liver fibrosis [16]. Interestingly, growing data also have proved the participation of Jnk, a critical protein of non-canonical Wnt pathway, in the progression of HBV-induced liver damage [17]. Thus, inhibition of non-canonical Wnt pathways may suppress HSC activation and liver fibrosis. Therefore, it is important to develop a compound that can suppress various non-canonical pathways simultaneously.

Notum, a secreted protease widely existed in living beings including humans, is a glypican-specific phospholipase [18]. More importantly, the currently accepted view is that Notum is a primarily a negative feedback inhibitor of Wnt signaling [19], which can deacylate all Wnt proteins and suppress various Wnt pathways concurrently [20–23]. In this study, we supposed that Notum can attenuate HBV-induced liver fibrosis through inhibiting Wnt 5a/NFAT and Wnt 5a/Jnk concurrently.

Wnt 5a has been regarded to induce various non-canonical Wnt pathways including NFAT and Jnk, respectively [24, 25]. In this study, Wnt 5a treatment significantly enhanced mRNA and protein expression of α-SMA and Col 1A1, up-regulated NFAT and pJnk protein expression in LX-2 cells, suggesting that Wnt 5a promoted HSC activation through both Wnt 5a/NFAT and Wnt 5a/Jnk pathways. On the contrary, Notum remarkably reduced mRNA and protein expression of α-SMA and Col 1A1, down-regulated NFATc1 and Jnk activation in LX-2 cells, indicating that the anti-fibrotic effect of Notum was related to the inhibition of Wnt 5a mediated NFATc1 and Jnk signaling pathways.

Hepatic stellate cells have been considered as the source of excessive deposition of ECM. In order to further investigate the anti-fibrotic effects of Notum, we detected the viability of LX-2 cells. Notum decreased the viability of LX-2 cells elevated by Wnt 5a treatment. However, Notum had no effect on LX-2 proliferation. These results lead to the conclusion that Notum attenuated liver fibrosis though decreasing hepatic stellate cell viability by down-regulating Wnt 5a mediated non-canonical Wnt pathways.

Hepatitis B virus remains a major health problem worldwide and it is still one of the main etiologies of liver fibrosis. In this study, we used Hep AD38, a stable HBV producing cell line, as our research model to explore the association of HBV replication and Wnt signaling. As shown in Fig. 2, ETV sharply reduced HBV DNA level in supernatant of Hep AD38 and inhibited HBV DNA replication in Hep AD38 cell in a time-dependant manner. Most importantly, inhibition of HBV replication lead to a significant reduction of Wnt 5a level both in supernatant and Hep AD38 cells. These results suggested that HBV replication enhanced Wnt 5a expression and secretion which accelerated HSC activation and liver fibrosis.

In order to further explore the mechanism of HBV induced liver fibrosis, we detected Wnt signaling activation and fibrosis gene expression in a cell co-culture model [26]. In this model, we could simulate cell–cell interaction in the process of liver fibrosis under the circumstance of HBV infection. Not surprisingly, as shown in Fig. 3, Hep AD 38 co-culture significantly increased mRNA and protein expressions of α-SMA, TIMP-1 and Col 1A1 as well as protein expressions of NFATc1 and pJnk in LX-2 cells compared with mock group, while they were all significantly reduced by Notum suggesting that Notum inhibited HBV induced liver fibrosis through down-regulating Wnt 5a mediated pathways.

In addition, we observed the association between Wnt 5a signaling pathway and liver fibrosis in HBV-infected patients. As shown in Fig. 4, with the progression of liver fibrosis, the expression of NFATc1 and pJnk in liver tissue increased. Not surprisingly, in Fig. 5a, mRNA expression of NFATc1, α-SMA, Col 1A1 and TIMP-1 in liver tissue increased significantly along with the development of liver fibrosis. Unfortunately, as shown in Fig. 5b, patients with HBV DNA > 10^5^ copies/ml had higher mRNA levels of NFATc1 and fibrosis genes in liver tissue than patients with HBV DNA < 10^5^ copies/ml, indicating that HBV contributed to liver fibrosis through activating Wnt pathway. Finally, both NFATc1 and pJnk protein expressions had positive correlations with liver fibrosis score (Fig. 5c), proving the involvement of Wnt 5a/NFATc1 and Wnt 5a/Jnk pathways in HBV-induced liver fibrosis.

**Fig. 6:**
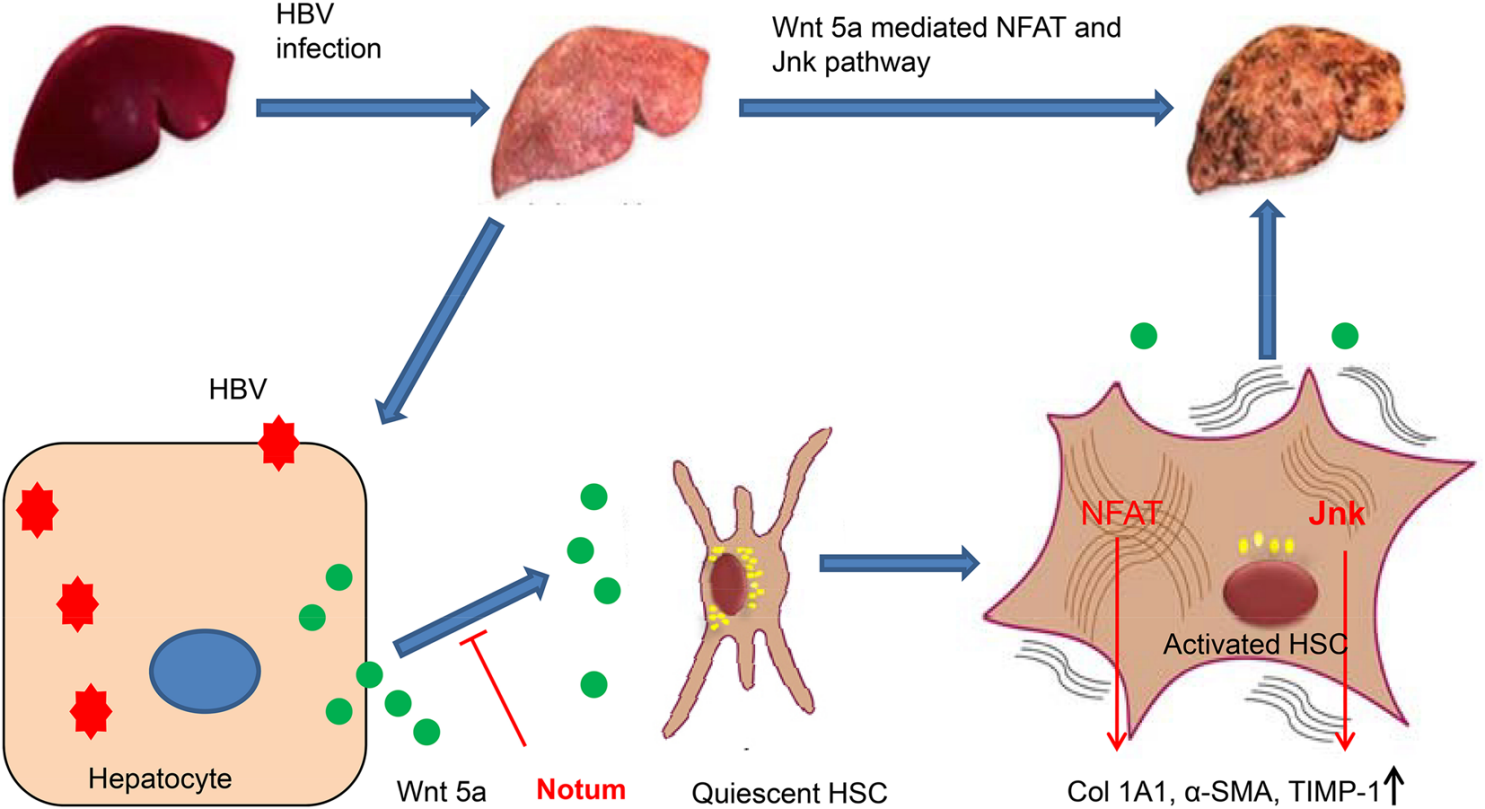
Proposed model by which HBV induced liver fibrosis through Wnt 5a mediated NFAT and Jnk pathway. HBV infection enhanced Wnt 5a production and secretion by hepatocyte, which in turn activated HSC by regulating NFAT and Jnk pathway. Notum can deacylate Wnt 5a protein, and subsequently inhibit NFATc1 and Jnk mediated HSC activation and liver fibrosis

In conclusion, these results demonstrated that Notum inhibited HBV related liver fibrosis through suppressing Wnt 5a mediated NFAT and Jnk pathways (Fig. 6). This study suggested Notum as a valuable target to fight HBV-induced liver fibrosis.

## Conclusions

We identified that HBV replication contributes to liver fibrosis through up-regulating Wnt 5a mediated non-canonical pathways, which can be inhibited by Notum. This founding may provide a new candidate for anti-fibrotic drug in patients with chronic HBV.

## Abbreviations

HBV: hepatitis B virus
α-SMA: α-smooth muscle actin
Col 1A1: collagen type 1 alpha 1
TIMP-1: tissue inhibitor of metalloproteinase 1
HSC: hepatic stellate cell
ECM: extracellular matrix
H&E: haematoxylin and eosin
ALT: alanine aminotransferase
AST: aspartate aminotransferase
TBil: total bilirubin
ALB: albumin
HRP: horseradish peroxidase
ANOVA: one-way analysis of variance
CHB: chronic hepatitis B
